# Graft Survival after Penetrating Keratoplasty in Cases of Trabeculectomy versus Ahmed Valve Implant

**DOI:** 10.1155/2018/9034964

**Published:** 2018-08-13

**Authors:** Abdelhamid Elhofi, Hany Ahmed Helaly

**Affiliations:** Ophthalmology Department, Faculty of Medicine, Alexandria University, Egypt

## Abstract

**Purpose:**

To compare the corneal graft survival rates after penetrating keratoplasty (PKP) in cases of post-PKP glaucoma managed by either trabeculectomy with mitomycin C or Ahmed glaucoma valve (AGV).

**Methods:**

This study was a retrospective interventional comparative study that included 40 eyes of 40 patients. The included patients had undergone previous PKP for anterior segment reconstruction after microbial or fungal keratitis, chemical burns, trauma, or perforated corneal ulcer. Post-PKP glaucoma was managed surgically by either trabeculectomy with mitomycin C (group 1) or Ahmed glaucoma valve (group 2).

**Results:**

The first group (*n*=20) had undergone trabeculectomy with MMC, and the second group (*n*=20) had undergone AGV implantation. Regarding BCVA, there was no statistically significant difference between the 2 groups. Mean IOP was significantly lower in the AGV group at 6 months, 12 months, and 24 months (*p*=0.001). Mean IOP at 24 months dropped significantly from preglaucoma surgery levels in both groups (*p*=0.001). Rejection episodes occurred in 2 eyes (10%) of the trabeculectomy group versus 8 eyes (40%) in the AGV group (*p*=0.028). In the trabeculectomy group, corneal graft failure occurred in 1 (5%), 3 (15%), and 6 (30%) eyes at 6 months, 12 months, and 24 months, respectively. In the AGV group, corneal graft failure occurred in 2 (10%), 5 (25%), and 10 (50%) eyes at 6 months, 12 months, and 24 months, respectively. The mean time to failure in the trabeculectomy group was 12.33 ± 5.60 months versus 11.90 ± 5.70 months in the AGV group (*p*=0.027).

**Conclusion:**

Managing postpenetrating keratoplasty glaucoma could be bothersome especially in complex cases. Ahmed glaucoma valve implant controls the intraocular pressure more effectively than trabeculectomy with mitomycin C. However, Ahmed glaucoma valve can result in higher rates of corneal graft failure in a shorter duration of time. This trial is registered with PACTR201712002861391 on 21 Dec 2017.

## 1. Introduction

Penetrating keratoplasty (PKP) is a common procedure for anterior segment reconstruction in cases of damaged corneas such as chemical burns, microbial keratitis, and perforated corneal ulcers [1, 2]. Lamellar keratoplasty in such conditions is of no use as it does not replace the damaged endothelium. In cases of preserved corneal endothelium, deep anterior lamellar keratoplasty may be successful for treating infectious keratitis or ocular burns. However, PKP carries higher risk of rejection and longer postoperative rehabilitation time than lamellar keratoplasty [3–5].

The reported incidence of raised intraocular pressure (IOP) and/or glaucoma after penetrating keratoplasty is variable according to the previous state of the eye before PKP. It was reported as low as 0–12% in PKP done for keratoconus and up to 75% in PKP done for infectious keratitis [6–9]. The pathogenesis of post-PKP glaucoma is multifactorial and may be due to postoperative inflammatory response, the formation of peripheral anterior synechiae (PAS), distortion of the trabecular meshwork, and previously undiagnosed glaucoma [6, 10].

The management of post-PKP glaucoma can be done using topical antiglaucoma medications, trabeculectomy with mitomycin C (MMC), deep sclerectomy, or glaucoma drainage device (GDD) [11–14]. Deep sclerectomy is valuable when the angle is not closed by synechiae and is associated with higher graft survival compared with trabeculectomy with MMC. The GDD may be valved, for example, Ahmed glaucoma valve, or nonvalved, for example, Molteno implant and Baerveldt implant [15–18]. An advantage of a valved implant such as Ahmed glaucoma valve is the low frequency of hypotony besides the easy insertion [19, 20]. However, it has higher rate of increased IOP in the first few months that may require needling and 5-fluorouracil injection [21].

The aim of the current study was to compare the corneal graft survival rates after penetrating keratoplasty (PKP) in cases of post-PKP glaucoma managed by either trabeculectomy with mitomycin C or Ahmed glaucoma valve.

## 2. Subjects and Methods

This study was a retrospective interventional comparative study that included 40 eyes of 40 patients. The included patients had undergone previous PKP for anterior segment reconstruction after microbial or fungal keratitis, chemical burns, trauma, or perforated corneal ulcer. Those patients developed uncontrolled IOP despite maximal medical therapy (i.e., 3 topical antiglaucoma medications), did not tolerate the medical therapy, or were not compliant. Post-PKP glaucoma was managed surgically by either trabeculectomy with mitomycin C (group 1) or Ahmed glaucoma valve (group 2). Included patients had a clear corneal graft before the glaucoma surgery, were >18 years of age, and had complete records of at least two years follow-up after glaucoma surgery. Data of the patients were recorded including best-corrected visual acuity (BCVA), IOP, clarity of the corneal graft, indication of the original PKP, corneal graft endothelial cell count, and any complications. Patients were recalled for a final follow-up visit. Snellen's BCVA was transformed into logMAR units. Counting fingers was considered as 2.1 logMAR, and hand motions was considered as 2.4 logMAR [22, 23].

The current study was approved by the local ethics committee of the faculty medicine, Alexandria University, Egypt. Tenets of the Declaration of Helsinki were followed. All patients signed an informed consent at the final follow-up visit.

## 3. Surgical Technique

Penetrating keratoplasty was performed under general anesthesia. Trephination was done using Hessburg–Barron trephines (Katena, Denville, USA). The donor graft was oversized by 0.25 mm larger than the recipient bed. Partial trephination of the recipient cornea was done using suction trephine size 7.5, 7.75, or 8 mm centered on the geometric center of the cornea, and cutting of the recipient cornea was completed by corneal scissors after full thickness trephination. For all patients, 10-0 nylon sutures (Alcon Laboratories, Fort Worth, Texas, USA) were applied. The donor cornea was initially secured in the recipient bed with four cardinal sutures at the 12, 6, 3, and 9 o'clock positions. Then the patients received 16 interrupted sutures. Any adhesions in the anterior segment were dissected, and cataract removal was done when applicable with or without intraocular lens implantation. Patients received topical gatifloxacin (Zymar, Allergan, Irvine, California, USA) every 6 hours for 30 days and topical prednisolone (Pred Forte, Allergan, Irvine, California, USA) every 6 hours tapered over 2 to 3 months and then replaced by topical fluorometholone (Flucon, Alcon Laboratories, Fort Worth, Texas, USA).

Trabeculectomy was performed under general anesthesia. Limbal-based conjunctival flap was dissected followed by dissection of a partial thickness triangular scleral flap. Application of a soaked sponge with 0.02% mitomycin C for 2 minutes above and under the scleral flap was done followed by a thorough wash with balanced salt solution. A paracentesis was done to test for aqueous drainage and to form the anterior chamber if needed. Then a corneoscleral block was excised and a peripheral iridectomy was performed. Closure of the scleral flap with interrupted 10-0 nylon sutures was done. The conjunctiva was closed with 10-0 running nylon sutures to form the filtration bleb. All surgeries were performed by the same surgeon (A.E.) with a reproducible technique.

Ahmed glaucoma valve (AGV) was performed under general anesthesia. Priming of the AGV using 26-gauge needle was done with injection of balanced salt solution to ensure functionality. A superior-temporal fornix based conjunctival flap was dissected between the 2 recti muscles. Tenon's capsule is dissected from the episclera. The body of AGV is placed 8–10 mm from the limbus and sutured with 10-0 nylon sutures to the sclera. The tube is then cut to allow 2–3 mm inside the anterior chamber and beveled up with an angle of 30°. Using 23-gauge needle, a tract is formed entering the anterior chamber parallel to the iris plane starting 1–3 mm posterior to the limbus. The beveled tube was then inserted through this tract avoiding contact with the iris or the corneal endothelium. All surgeries were performed by the same surgeon (A.E.) with a reproducible technique.

Graft failure was defined as corneal edema for 1 month or more despite the use of intense steroid therapy or irreversible corneal graft opacity as a result of scarring or neovascularization. Time to failure was defined as the time interval between the glaucoma surgery and the diagnosis of graft failure according to the previous criteria. Glaucoma surgery was considered successful if the IOP was ≤21 mmHg with (qualified success) or without (complete success) topical medications and/or needling with subconjunctival 5-fluorouracil.

Data analysis was performed using the software SPSS for Windows version 20.0 (SPSS Inc., Chicago, USA). Quantitative data were described using range, mean, and standard deviation. The Mann–Whitney test was used to compare means of independent samples. Wilcoxon rank-sum test was used for comparisons between means of the preoperative and postoperative data. The Kruskal–Wallis test was used to compare means of two or more groups. Kaplan–Meier was used for survival analysis. The chi-square test was used to compare between different percentages and ratios. Differences were considered statistically significant when the associated *p*-value was less than 0.05.

## 4. Results

Forty post-PKP glaucoma patients were included and divided into two equal groups. The first group (*n*=20) had undergone trabeculectomy with MMC, and the second group (*n*=20) had undergone AGV implantation.  Table 1 summarizes the characteristics of the included eyes of both groups. Microbial or fungal keratitis was the main indication for PKP in both groups. Using Mann–Whitney and chi-square tests, there were no statistically significant differences between the 2 groups regarding different parameters. Before the glaucoma surgery, the eyes of the Ahmed glaucoma valve group had higher mean IOP and lower post-PKP endothelial cell count (ECC), but this was not statistically significant. Gonioscopy was done to evaluate the degree of PAS. Total PAS more than 270° was found in 11 versus 13 eyes in groups 1 and 2, respectively. Partial PAS less than 180° was found in 6 versus 5 eyes in groups 1 and 2, respectively. None of the included cases had a history of previous glaucoma.

Table 2 shows the characteristics of the included eyes of both groups after the glaucoma surgery. Regarding BCVA, there was no statistically significant difference between the 2 groups. Also, there is no statistically significant difference between the preoperative mean BCVA and 24-month postoperative mean BCVA in both groups (*p*=0.511and0.532 in groups 1 and 2, respectively). Two patients from each group have lost 2 lines of BCVA after the glaucoma surgery. Mean IOP was significantly lower in the AGV group at 6 months, 12 months, and 24 months (*p*=0.001). Mean IOP at 24 months dropped significantly from preglaucoma surgery levels in both groups (*p*=0.001). Regarding mean number of antiglaucoma medications used, there is a significant decrease after the glaucoma surgery from preoperative levels. It was significantly lower in the AGV group at 24 months (*p*=0.034) but not at 6 and 12 months (*p*=0.157, 0.102, respectively). At 24 months, six patients of the trabeculectomy group received one topical antiglaucoma medication, and 4 patients received 2 medications. While in the AGV group, six patients received 1 medication, and 1 patient received 2 medications.

The needling procedure with subconjunctival 5-fluorouracil injection was needed in a total of 14 eyes (70%) of the trabeculectomy group versus 6 eyes (30%) in the AGV group (*p*=0.011) at 24 months. The total success rate (complete and qualified) was higher in the AGV group than the trabeculectomy group. This difference was statistically significant at 12 and 24 months (*p*=0.048  and  0.001, respectively) but not at 6 months (*p*=0.179).

Regarding postoperative complications, two cases had vitreous hemorrhage in the trabeculectomy group, and none developed hypotony or choroidal effusion. Cases with aphakia needed anterior vitrectomy to prevent vitreous clogging. In the AGV group, one case developed vitreous hemorrhage in the early postoperative period that persisted after pars plana vitrectomy with failed 3 times needling and subconjunctival 5-fluorouracil to control IOP. Cyclocryodestruction was needed to control IOP in this case. Two other cases developed vitreous hemorrhage that resolved spontaneously with no further intervention. One case had early tube obstruction due to a vitreous strand and required anterior vitrectomy. Three cases had tube-related complications. One case had a small conjunctival hole that was managed conservatively using topical tetracycline ointment. Another case developed larger exposure of the implant which required a scleral patch graft. Another case had tube extrusion from the anterior chamber and required a tube extensor with a scleral patch graft. By the end of 2nd year of follow-up, seven cases of the trabeculectomy group required another glaucoma surgery in the form of Ahmed glaucoma valve implantation versus three cases in the AGV group.

**Figure 1:**
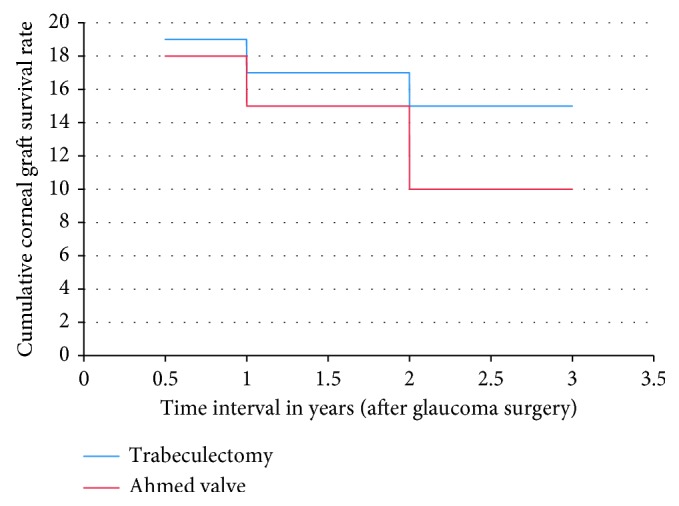
Kaplan–Meier survival analysis for corneal graft survival among trabeculectomy and Ahmed valve groups.

Rejection episodes occurred in 2 eyes (10%) of the trabeculectomy group versus 8 eyes (40%) in the AGV group (*p*=0.028). Among the 8 eyes of the AGV group, one eye had 3 rejection episodes, four eyes had 2 rejection episodes, and three eyes had 1 rejection episode. In the trabeculectomy group, corneal graft failure occurred in 1 (5%), 3 (15%), and 6 (30%) eyes at 6 months, 12 months, and 24 months, respectively. In the AGV group, corneal graft failure occurred in 2 (10%), 5 (25%), and 10 (50%) eyes at 6 months, 12 months, and 24 months, respectively. However, this difference was not statistically significant (*p*=0.902).  Figure 1 shows Kaplan–Meier survival analysis graph for the cumulative corneal graft survival in both groups. The mean time to failure in the trabeculectomy group was 12.33 ± 5.60 months (range 4–18 months). The mean time to failure in the AGV group was 11.90 ± 5.70 months (range 3–18 months). There was a statistically significant difference between the two groups (*p*=0.027). Cases with graft failure required another PKP. One case in the AGV group required two PKP surgeries.

## 5. Discussion

Postpenetrating keratoplasty glaucoma is not uncommon. It is the second leading cause for corneal graft failure. Post-PKP glaucoma is a problematic issue because of difficulties met in diagnosis and management. The increase in IOP may have damaging effect on the corneal endothelial cells and therefore the corneal graft failure. Early diagnosis of post-PKP rise of IOP is essential in preserving the integrity of optic nerve head and corneal graft clarity [24, 25].

In the current study, the incidence of post-PKP glaucoma was high (more than 50%). This is due to the complex nature of the indication for PKP of the included patients. About half of the cases had microbial or fungal keratitis, and one quarter had chemical burns. Many of those patients developed PAS. Other possible mechanisms may be due to postoperative steroid medications used, trabecular meshwork damage either due to the original disease or due to anterior chamber collapse, or postoperative inflammatory response [6, 10]. As stated above, the incidence of post-PKP glaucoma varies according to the indication of PKP. Yildirim et al. [26] reviewed 122 eyes of PKP. Post-PKP glaucoma occurred in 42 eyes which represented 34% of the cases during the first 4 years of follow-up. They reported a mean time interval from PKP to the diagnosis of post-PKP glaucoma of 24 weeks. The difference in incidence rate from our study may be related to the different indications for PKP. The longer the duration of follow-up, it is expected to have higher incidence of post-PKP glaucoma.

In the current study, all cases of trabeculectomy surgery included the application of 0.02% of mitomycin C for 2 minutes in the subconjunctival and subscleral spaces. Mitomycin C application significantly improved the success rates of trabeculectomy for managing post-PKP glaucoma. Mitomycin C should be thoroughly washed before the entry of the anterior chamber to avoid endothelial cell damage. The reported success rates of IOP control in such patients vary from 67 to 90% [26, 27]. In the current study, the success rate for the trabeculectomy group was 90% at 6 months postoperative which dropped to 55% at the end of the second year. It is important during the surgery to take care to prevent collapse or shallowing of the anterior chamber to decrease the damage to the endothelial cells. The use of 5-fluorouracil for subconjunctival injection may be of benefit with needling procedure, but it is associated with corneal epithelial toxicity. The incidence of corneal graft failure in the current study was 5% at 6 months postoperative and reached 30% at the end of 2 years follow-up. Some studies reported the rate of graft failure after trabeculectomy for post-PKP glaucoma to be 12–18% [26–30].

The success rate of IOP control in the Ahmed glaucoma valve group was 95% at 6 months and 80% at 2 years. This was significantly higher than the trabeculectomy group. Rejection episodes occurred at a higher incidence with the AGV group. Corneal graft rejection occurred in 10% at 6 months and in 50% at 2 years. However, this difference failed to show statistical significance despite of the clinical significance. It may be resorted to the smaller number of the included patients due to the search for more complicated cases to be included in the study. The mean time to failure was significantly shorter in the AGV group. Kirkness [31] was the first one to report the use of a glaucoma drainage device for the management of post-PKP glaucoma in the late 80's. Ahmed glaucoma valve has an advantage over the nonvalved devices being easy to insert and having a lower incidence of postoperative hypotony. On the opposite, it is associated with higher incidence of postoperative rise of intraocular pressure that might indicate the use of needling and subconjunctival 5-fluorouracil [32, 33]. After 2 years follow-up, the included AGV group in the current study needed less postoperative needling than the trabeculectomy group (30% versus 70%). Ahmed glaucoma valve and other glaucoma drainage devices in general control IOL in a high percentage of the reported population (average of 84%) [32–35] which is comparable with the results of our study despite the complex nature of the included patients. However, they appear to have a higher incidence of corneal graft failure (average of 36%) [32–34]. Again, the incidence of graft failure in our AGV study group was higher due to the complex nature of the included cases and also due to the higher damage to the graft endothelium induced by the AGV compared with trabeculectomy. Helmy et al. [35] reported a graft survival rate of 94% at 4 years follow-up in their series of 38 eyes of post-PKP glaucoma managed by Ahmed valve. The cause of increased incidence of corneal graft failure after glaucoma drainage device may be related to the retrograde passage of inflammatory cells into the anterior chamber, postoperative inflammation that breaks down the blood aqueous barrier, formation of peripheral anterior synechiae, and shallow anterior chamber with iris or tube endothelial touch.

The advantages of the current study are the comparative nature of two study groups with two different surgeries, relatively moderate follow-up period of 2 years, and focusing on selecting complex cases requiring anterior segment reconstruction with extensive PAS. The limitations of the current study included the need for larger number of included patients. However, the complex cases are not available in larger number. Also, it might have been useful to include a longer duration of follow-up to record the long-term effects.

In conclusion, managing postpenetrating keratoplasty glaucoma could be bothersome especially in complex cases. Ahmed glaucoma valve implant controls the intraocular pressure more effectively than trabeculectomy with mitomycin C. However, Ahmed glaucoma valve can result in higher rates of corneal graft failure in a shorter duration of time.

## Figures and Tables

**Table 1:** Characteristics of the included patients of trabeculectomy and Ahmed glaucoma valve groups before the glaucoma surgery.

	Trabeculectomy group (*n*=20)	Ahmed valve group (*n*=20)	*p* value^*∗*^
Age (years)	37.3 ± 9.6 (19–60)	35.7 ± 11.2 (20–61)	0.521

Sex (male : female)	11 : 9	10 : 10	0.752

Diabetes mellitus	2 (10%)	2 (10%)	1.000

Hypertension	5 (25%)	4 (20%)	0.705

Indication of keratoplasty			0.739
Microbial or fungal keratitis	9 (45%)	8 (40%)	
Chemical burn	5 (25%)	7 (35%)	
Trauma	4 (20%)	2 (10%)	
Others	2 (10%)	3 (15%)	

Pre-op BCVA (logMAR)	1.40 ± 0.55 (0.9–2.4)	1.30 ± 0.50 (0.8–2.1)	0.655

Pre-op IOP (mmHg)	31.22 ± 4.91 (26–40)	33.54 ± 4.59 (29–42)	0.375

Pre-op number of medications	2.11 ± 0.55	2.34 ± 0.61	0.410

Pre-op endothelial count (cells/mm^2^)	2812 ± 270 (2400–3210)	2776 ± 291 (2450–3120)	0.288

Pre-op lens status			0.881
Phakic	4	3	
Pseudophakic	12	12	
Aphakic	4	5	

Time interval from PKP to glaucoma surgery (months)	9.41 ± 7.22 (2–30)	8.66 ± 6.51 (2–28)	0.321

^*∗*^Using the Mann–Whitney test or the chi-square test where appropriate.

**Table 2:** Characteristics of the included patients of trabeculectomy and Ahmed glaucoma valve groups after the glaucoma surgery.

	Trabeculectomy group (*n*=20)	Ahmed valve group (*n*=20)	*p* value^*∗*^
*Post-op BCVA (logMAR)*			
6 months	1.21 ± 0.65 (0.7–2.4)	1.27 ± 0.56 (0.8–2.1)	0.491
12 months	1.29 ± 0.67 (0.8–2.1)	1.33 ± 0.60 (0.9–2.1)	0.544
24 months	1.35 ± 0.59 (0.8–2.1)	1.36 ± 0.64 0.9–2.1)	0.697

*Post-op IOP (mmHg)*			
6 months	13.20 ± 4.51 (8–21)	11.20 ± 3.32 (5–20)	0.001^#^
12 months	13.50 ± 4.76 (8–25)	11.88 ± 3.55 (7–22)	0.001^#^
24 months	13.98 ± 3.44 (10–26)	12.28 ± 3.71 (8–23)	0.001^#^

*Post-op number of medications*			
6 months	0.25 ± 0.44	0.15 ± 0.37	0.157
12 months	0.50 ± 0.76	0.25 ± 0.44	0.102
24 months	0.70 ± 0.80	0.40 ± 0.60	0.034^#^

*Eyes required needling + 5-fluorouracil*			
6 months	5 (25%)	2 (10%)	0.212
12 months	10 (50%)	4 (20%)	0.047^#^
24 months	14 (70%)	6 (30%)	0.011^#^

*Total success rate*			
6 months	90%	95%	0.179
12 months	80%	90%	0.048^#^
24 months	55%	80%	0.001^#^

^*∗*^Using the Mann–Whitney test or the chi-square test where appropriate. ^#^Significant.

## Abbreviations

PKP: Penetrating keratoplasty
IOP: Intraocular pressure
PAS: Peripheral anterior synechia
MMC: Mitomycin C
BCVA: Best-corrected visual acuity
AGV: Ahmed glaucoma valve.

## References

[1] Price M. O., Calhoun P., Kollman C., Price F. W., Lass J. H. (2016). Descemet stripping endothelial keratoplasty: ten-year endothelial cell loss compared with penetrating keratoplasty. *Ophthalmology*.

[2] Coster D. J., Lowe M. T., Keane M. C., Williams K. A., Contributors A. C. (2014). A comparison of lamellar and penetrating keratoplasty outcomes: a registry study. *Ophthalmology*.

[3] Romano V., Iovieno A., Parente G., Soldani A. M., Fontana L. (2015). Long-term clinical outcomes of deep anterior lamellar keratoplasty in patients with keratoconus. *American Journal of Ophthalmology*.

[4] MacIntyre R., Chow S. P., Chan E., Poon A. (2014). Long-term outcomes of deep anterior lamellar keratoplasty versus penetrating keratoplasty in Australian keratoconus patients. *Cornea*.

[5] Steven P., Siebelmann S., Hos D., Bucher F., Cursiefen C. (2017). Immune reactions and dry eye after Posterior Lamellar keratoplasty. *Proceedings of Current Treatment Options for Fuchs Endothelial Dystrophy*.

[6] Oruçoglu F., Blumenthal E. Z., Frucht-Pery J., Solomon A. (2014). Risk factors and incidence of ocular hypertension after penetrating keratoplasty. *Journal of Glaucoma*.

[7] Borderie V. M., Loriaut P., Bouheraoua N., Nordmann J. P. (2016). Incidence of intraocular pressure elevation and glaucoma after lamellar versus full-thickness penetrating keratoplasty. *Ophthalmology*.

[8] Quek D. T., Wong C. W., Wong T. T. (2015). Graft failure and intraocular pressure control after keratoplasty in iridocorneal endothelial syndrome. *American Journal of Ophthalmology*.

[9] Chen H. C., Lee C. Y., Lin H. Y. (2017). Shifting trends in microbial keratitis following penetrating keratoplasty in Taiwan. *Medicine*.

[10] Dada T., Aggarwal A., Minudath K. B. (2008). Post-penetrating keratoplasty glaucoma. *Indian Journal of Ophthalmology*.

[11] Doyle J. W., Smith M. F. (1994). Glaucoma after penetrating keratoplasty. *Seminars in Ophthalmology*.

[12] Beiran I., Rootman D. S., Trope G. E., Buys Y. M. (2000). Long-term results of transscleral Nd: YAG cyclophotocoagulation for refractory glaucoma postpenetrating keratoplasty. *Journal of Glaucoma*.

[13] Banitt M., Lee R. K. (2009). Management of patients with combined glaucoma and corneal transplant surgery. *Eye*.

[14] Panda A., Prakash V. J., Dada T., Gupta A. K., Khokhar S., Vanathi M. (2011). Ahmed glaucoma valve in post-penetrating-keratoplasty glaucoma: a critically evaluated prospective clinical study. *Indian Journal of Ophthalmology*.

[15] Lee R. K., Fantes F. (2003). Surgical management of patients with combined glaucoma and corneal transplant surgery. *Current Opinion in Ophthalmology*.

[16] Levinson J. D., Giangiacomo A. L., Beck A. D. (2015). Glaucoma drainage devices: risk of exposure and infection. *American Journal of Ophthalmology*.

[17] Muir K. W., Lim A., Stinnett S., Kuo A., Tseng H., Walsh M. M. (2014). Risk factors for exposure of glaucoma drainage devices: a retrospective observational study. *BMJ Open*.

[18] Chen A., Yu F., Law S. K., Giaconi J. A., Coleman A. L., Caprioli J. (2015). Valved glaucoma drainage devices in pediatric glaucoma: retrospective long-term outcomes. *JAMA Ophthalmology*.

[19] Li Z., Zhou M., Wang W. (2014). A prospective comparative study on neovascular glaucoma and non-neovascular refractory glaucoma following Ahmed glaucoma valve implantation. *Chinese Medical Journal*.

[20] Jiménez-Román J., Gil-Carrasco F., Costa V. P. (2016). Intraocular pressure control after the implantation of a second Ahmed glaucoma valve. *International Ophthalmology*.

[21] Al-Omairi A. M., Al Ameri A. H., Al-Shahwan S. (2017). Outcomes of Ahmed glaucoma valve revision in pediatric glaucoma. *American Journal of Ophthalmology*.

[22] Jackson T. L., Donachie P. H., Sparrow J. M., Johnston R. L. (2013). United Kingdom National Ophthalmology Database study of vitreoretinal surgery: report 1; case mix, complications, and cataract. *Eye*.

[23] Jackson T. L., Donachie P. H., Sallam A., Sparrow J. M., Johnston R. L. (2014). United Kingdom National Ophthalmology Database study of vitreoretinal surgery: report 3, retinal detachment. *Ophthalmology*.

[24] Wilson S. E., Kaufman H. E. (1990). Graft failure after penetrating keratoplasty. *Survey of Ophthalmology*.

[25] Goldberg D. B., Schanzlin D. J., Brown S. I. (1981). Incidence of increased intraocular pressure after keratolasty. *American Journal of Ophthalmology*.

[26] Yildirim N., Gursoy H., Sahin A., Ozer A., Colak E. (2011). Glaucoma after penetrating keratoplasty: incidence, risk factors, and management. *Journal of ophthalmology*.

[27] Chowers I., Ticho U. (1999). Mitomycin-C in combined or two stage procedure trabeculectomy followed by penetrating keratoplasty. *Journal of Glaucoma*.

[28] Ayyala R. S., Pieroth L., Vinals A. F. (1998). Comparison of mitomycin C trabeculectomy, glaucoma drainage device implantation and laser neodymium YAG cyclophotocoagulation in the management of intractable glaucoma after penetrating keratoplasty. *Ophthalmology*.

[29] Raj A., Dhasmana R., Bahadur H. (2017). Comparative evaluation of trabeculectomy with releasable suture versus subconjunctival mitomycin C in post keratoplasty glaucoma. *Sudanese Journal of Ophthalmology*.

[30] Iverson S. M., Spierer O., Papachristou G. C. (2015). Comparison of primary graft survival following penetrating keratoplasty and descemet’s stripping endothelial keratoplasty in eyes with prior trabeculectomy. *British Journal of Ophthalmology*.

[31] Kirkness C. M. (1987). Penetrating keratoplasty, glaucoma and silicon drainage tubing. *Developments in Ophthalmology*.

[32] Parihar J. K., Jain V. K., Kaushik J., Mishra A. (2017). Pars plana-modified versus conventional Ahmed glaucoma valve in patients undergoing penetrating keratoplasty: a prospective comparative randomized study. *Current Eye Research*.

[33] Ayyala R. S., Zurakowski D., Smith J. A. (1998). A clinical study of Ahmed valve implant in advanced glaucoma. *Ophthalmology*.

[34] Tandon A., Espandar L., Cupp D., Ho S., Johnson V., Ayyala R. S. (2014). Surgical management for postkeratoplasty glaucoma: a meta-analysis. *Journal of Glaucoma*.

[35] Helmy H., Hashem O., Abouelkhir S., Murad M. M. (2016). Ahmed valve implantation in post-penetrating keratoplasty glaucoma: four-years follow up. *Cataract and Cornea: Journal of the Egyptian Society of Cataract and Corneal Diseases*.

